# Evaluation of Shiraz wastewater treatment plant effluent quality for agricultural irrigation by Canadian Water Quality Index (CWQI)

**DOI:** 10.1186/1735-2746-10-27

**Published:** 2013-04-08

**Authors:** Mohammad A Baghapour, Simin Nasseri, Babak Djahed

**Affiliations:** 1 Department of Environmental Health Engineering, School of Health and Nutrition, Shiraz University of Medical Sciences, Shiraz, Iran; 2 Department of Environmental Health Engineering, School of Public Health and Institute of Public Health Research, Tehran University of Medical Sciences, Tehran, Iran; 3 Department of Environmental Health Engineering, School of Health and Nutrition, Shiraz University of Medical Sciences, Shiraz, Iran

**Keywords:** Effluent, Water reuse, Irrigation, CWQI, Shiraz

## Abstract

**Background:**

Using treated wastewater in agriculture irrigation could be a realistic solution for the shortage of fresh water in Iran, however, it is associated with environmental and health threats; therefore, effluent quality assessment is quite necessary before use. The present study aimed to evaluate the physicochemical and microbial quality of Shiraz wastewater treatment plant effluent for being used in agricultural irrigation. In this study, 20 physicochemical and 3 microbial parameters were measured during warm (April to September) and cold months (October to march). Using the measured parameters and the Canadian Water Quality Index, the quality of the effluent was determined in both warm and cold seasons and in all the seasons together.

**Results:**

The calculated index for the physicochemical parameters in the effluent was equal (87) in warm and cold months and it was obtained as 85 for the seasons all together. When the microbial parameters were used in order to calculate the index, it declined to 67 in warm and cold seasons and 64 in all the seasons together. Also, it was found that three physicochemical parameters (TDS, EC, and NO_3_) and three microbial parameters (Fecal coliform, Helminthes egg, and Total coliform) had the most contribution to the reduction of the index value.

**Conclusions:**

The results showed that the physicochemical quality of Shiraz Wastewater Treatment Plant Effluent was good for irrigation in the warm, cold, and total of the two kinds of seasons. However, by applying the microbial parameter, the index value declined dramatically and the quality of the effluent was marginal.

## Background

The Middle East and North Africa countries (MENA), with one percent of fresh water resources, are the most arid regions of the world [1]. Due to the scarcity of fresh water resources in these regions, wastewater reuse could be a realistic option to alleviate the shortage of fresh water resources in these communities and until now, the largest and most popular wastewater reuse has been in the agricultural irrigation field [2,3]. Wastewater reuse is not a new issue; for instance, indications of wastewater reuse for agricultural irrigation extends back about 3000 years to the Minoan Civilization in Greece [4]. Also, the history of wastewater reuse in Iran is related to the Safavieh era (1501-1722AD) [5]. The main advantages of using the municipal Wastewaters Treatment Plants Effluent (WWTPE) are availability, being inexpensive to irrigate farmland, and being a constant source of fresh water [6,7]. Other benefits of wastewater reuse are the possibility to recover the nutrients in the wastewater, reducing the use of fertilizers [6,8-11], resolving the problems associated with wastewater disposal [10-12], and groundwater recharge [10]. So today, there are plans for the wastewater reuse in many countries; for example, in Spain, using wastewater for irrigation is about 346 MCM/year and amount of wastewater reuse could be 1100 hm^3^ by 2012 [12,13]. In California, about 78% of the treated wastewater is used for agricultural irrigation in central Valley and coastal areas [8]. Moreover, it is estimated that the treated wastewater effluent could be the main (about 70%) source of water for irrigation in Israel by 2040 [2]. Nevertheless, since some materials remain in wastewater effluent, despite the above-mentioned benefits, wastewater reuse could be associated with some risks [14]. Thus, several studies have evaluated the probable health and environmental impacts of wastewater reuse in agricultural irrigation. For example, in separate studies, Surdyk et al. [15], Wang et al. [16], and Reboll et al. [17] concluded that irrigation with wastewater effluent had no negative impacts on various agricultural products. However, in some studies evaluating the long-term effects of the wastewater effluent on the soil, the heavy metal pollution and reduction of soil quality have been reported [11,18]. On the other hand, due to the presence of pathogens in wastewater effluent, the irrigation by this water resource could be associated with health hazards and increasing the risk of intestinal infections [19,20]. Therefore, it seems that quality assessment of wastewater effluent before reuse projects is essential in order to prevent adverse health and environmental impacts.

According to the indicators of UN and the International Water Management Institute (IWMI), Iran is in a severe water crisis situation [21]. Thus, using new fresh water resources is very important in this country, especially in Fars province (in the southwest of Iran) in which, drought is considered as the main climatic feature [22]. Overall, it seems that Shiraz Wastewater Treatment Plant Effluent (SWTPE) could potentially be considered as a good source of fresh water supply and Fars Regional Water Organization plans to reuse SWTPE (about 29.5 MCM/year); hence, the current study aims to evaluate SWTPE quality for agricultural irrigation.

## Materials and Methods

### Status of the Shiraz wastewater treatment plant

SWTP is located in the southeastern region of the city. It covers 409000 inhabitants right now and it is estimated that the final coverage of inhabitants in this WWTP will be about 548000 in future. The average inlet flow rate of this WWTP is about 930 LPS and it is expected to provide about 29.5MCM/year of fresh water for irrigation. Activated sludge is the biological wastewater treatment processes of this WWTP and it includes different units of screen bar unit, primary settling tank, selector, aerated tank, secondary settling tank, and chlorination unit.

### Sampling and measured parameters

In order to determine the quality of the SWTP for being reused in the agricultural irrigation, 20 physicochemical and 3 microbial parameters were evaluated during warm (April to September) and cold months (October to march). Then, 11 samples in warm and 7 samples in cool seasons were taken and analyzed from effluent of WWTP (grab sampling was used). The measured physiochemical parameter were pH, EC, TSS, TDS, Res.Cl, HCO_3_, Cl, SO_4_, Ca, Mg, Na, Mn, Hg, Fe, As, Cd, DO, COD, BOD_5_, and NO_3_, while the 3 microbial parameters included Fecal coliform, Total coliform, and Helminthes egg. It is worth noting that due to some limitations, helminthes egg and SO_4_ were measured just 8 (4 times in warm seasons and 4 times in cold seasons) and 12 times (6 times in warm seasons and 6 times in cold seasons), respectively.

### Apparatus

The EC and pH of the study samples were measured using EC meter Metrohm (model 856) and pH Meter metrohm (model 827). In addition, the amounts of Ca, Mg, and Na were measured by Flame photometer Jenway (model PFP7). In order to measure COD and SO_4_, Spectrophotometer HACH (model DR/2500) was used. Also, by an Atomic Absorption Spectrometer GBC Scientific Equipment (model savant AA AAS), the concentrations of Mn, Fe, Hg, As, and Cd were determined in the samples. The concentration of DO in SWTP effluent was measured by DO meter HACH (model 850045). Also, Spectrophotometer PG Instruments Ltd (model T80) and Manometric respirometer HACH (model BOD Trak II) were used in order to measure NO_3_ and BOD_5_, respectively. Finally, Nickon microscope (model E100) was used for counting the number of helminthes egg.

### Determination of the effluent quality

In order to determine the quality of the SWTPE, Canadian Water Quality Index (CWQI) was used. In general, three factors (F_1_, F_2_, and F_3_) are used to determine the CWQI. F_1_ (scope) indicates the percentage of the variables which depart from their objectives (Eq. (1)), while F_2_ (Frequency) represents the percentage of the tests which do not meet the objectives (Eq. (2)) [23,24].

(1) $$F_{1}=\left(\frac{\mathrm{Number}\;\mathrm{of}\;\mathrm{failed}\;\mathrm{varialbes}}{\mathrm{Total}\;\mathrm{number}\;\mathrm{of}\;\mathrm{varialbes}}\right)\times 100$$

(2) $$F_{2}=\left(\frac{\mathrm{Number}\;\mathrm{of}\;\mathrm{failed}\;\mathrm{varialbes}}{\mathrm{Total}\;\mathrm{number}\;\mathrm{of}\;\mathrm{varialbes}}\right)\times 100$$

F_3_ (Amplitude) is calculated by an asymptotic capping function which scales the normalized sum of the excursions from the objectives (nse) in a range between 0 and 100 (Eq. (3)). F_3_ is obtained in a three-step process. At the first step, the "excursion" is calculated and the number of times an individual parameter is further than (when the objective is a minimum, less than) the objective is nominated as “excursion” and is calculated by Eq. (4) and Eq. (5). (In case the test value should not fall below the objective, Eq. (5) is used).

(3) $$F_{3}=\frac{\mathrm{nse}}{0.01\mathrm{nse}\;+\;0.01}$$

(4) $$\mathrm{excursio}n_{i}=\left(\frac{\mathrm{Failed}\;\mathrm{Test}\;\mathrm{Valu}e_{i}}{\mathrm{Objectiv}e_{i}}\right)-1$$

(5) $$\mathrm{excursio}n_{i}=\left(\frac{\mathrm{Objectiv}e_{i}}{\mathrm{Failed}\;\mathrm{Test}\;\mathrm{Valu}e_{i}}\right)-1$$

(6) $$\mathrm{nse}=\frac{\sum_{i=1}^{n}\mathrm{excursio}n_{i}}{\mathrm{number}\;\mathrm{of}\;\mathrm{test}}$$

Then, the sum of the excursions from the objectives is calculated by Eq. (6) and, finally, the CWQI could be obtained from Eq. (7). It should be noted that 1.732, is a scaling factor and rearranges the index between 0 and 100 [25].

(7) $$\mathrm{CWQI}=100-\left(\frac{\sqrt{F_{1}^{2}+F_{2}^{2}+F_{3}^{2}}}{1.732}\right)$$

Different values obtained from the CWQI are classified in Table 1.

**Table 1:** **Classification of CWQI values**[26]

**Rank**	**WQI value**	**Description**
Excellent	95-100	There is no threat to the water quality and these index values can only be obtained when all parameters are within objectives virtually all the time.
Very Good	89-94	There is a slight presence of threat or impairment for the water quality
Good	80-88	There is minor degree of threat for the water quality; conditions rarely depart from desirable levels.
Fair	65-79	Water quality is usually protected but occasionally threatened; sometimes conditions depart from desirable conditions
Marginal	45-64	Water quality is frequently threatened; conditions often depart from natural or desirable levels.
Poor	0-44	Water quality is almost always threatened andconditions usually depart from desirable levels.

The objectives used in the present study were selected based on the Iranian Department of Environment (IDOE) standards for wastewater reuse in agricultural irrigation; however, due to the lack of IDOE standards in this field, WHO, USEPA, and Jordan standards were used (Table 2). Also, the 90% cumulative probability was calculated for all the parameters and compared with the standards. Furthermore, since the effect of sodium should be considered in association with calcium and magnesium, Sodium Adsorption Ratio was used (SAR) instead of Na for calculating the CWQI.

**Table 2:** Minimum, maximum, mean, and cumulative probability of each measured parameter

**Parameter**	**Unit**	**Min**	**Max**	**Mean**	**Cumulative probability (less than90%)**	**Standard**	**Specific Multiplier**	**Contribution Value %**
pH (Warm)	-	7.46	8.25	7.861±0.281	7.57	6.5-8.5 (Iran)	0.5	3.88
pH (Cool)		7.69	8.17	7.902±0.192	8.181			
pH (Overall)		7.46	8.25	7.877±0.057	8.22			
EC (Warm)	μmoh/cm	1717	2351	1904±189.208*	2100*	700 (WHO)	0.1	0.78
EC (Cool)		1722	2340	1928.14±221.977*	2343*			
EC (Overall)		1717	2351	1913.39±46.306*	2340*			
TSS (Warm)	mg/L	18	115	61.18±33.722	80	100 (Iran)	0.5	3.88
TSS (Cool)		15	163	69.57±49.027	165			
TSS (Overall)		15	163	64.44±9.234	115			
TDS (Warm)	mg/L	1144	1518	1269.36±105.766*	1365*	450 (WHO)	1	7.76
TDS (Cool)		1126	1530	1311.83±165.153*	1533*			
TDS (Overall)		1126	1530	1284.35±30.633*	1518*			
Res. Cl (Warm)	mg/L	0	0	0±0	0	0.2 (Iran)	0.5	3.88
Res. Cl (Cool)		0	0.25	0.057±0.101	0.26			
Res. Cl (Overall)		0	0.25	0.022±0.015	0.15			
HCO_3_ (Warm)	mg/L	365.94	542.811	441.345±58.134	540.523	520 (Jordan)	0.5	3.88
HCO_3_ (Cool)		378.138	518.415	424.316±54.987	523.315			
HCO_3_ (Overall)		365.94	542.811	434.723±13.182	518.415			
Cl (Warm)	mg/L	248.171	372.256	283.624±33.596	369.564	600 (Iran)	0.5	3.88
Cl (Cool)		219.808	301.35	271.721±27.418	308.593			
Cl (Overall)		219.808	372.256	278.995±7.321	301.35			
SO_4_ (Warm)	mg/L	171.465	265.65	224.112±44.357	262.863	1000 (Jordan)	0.5	3.88
SO_4_ (Cool)		182.091	444.36	256.473±100.383	500			
SO_4_ (Overall)		171.465	444.36	240.292±21.909	444.36			
Ca (Warm)	mg/L	100.2	130.26	114.956±8.129	127.35	200 (EPA)	0.5	3.88
Ca (Cool)		94.188	180.36	116.518±30.555	195			
Ca (Overall)		94.188	180.36	115.564±4.527	130.26			
Mg (Warm)	mg/L	54.675	91.125	71.795±10.444	90.85	100 (Iran)	0.5	3.88
Mg (Cool)		30.375	100.845	67.345±23.568	101.92			
Mg (Overall)		30.375	100.845	70.065±3.838	91.125			
SAR (Warm)	-	2.677	5.156	3.405±0.830	4.7	9 (FAO)	1	7.76
SAR (Cool)		2.897	5.087	3.63±0.840	5.3			
SAR (Overall)		2.677	5.156	3.493±0.192	5.087			
Mn (Warm)	mg/L	0.0062	0.042	0.02±0.011	0.041	1 (Iran)	1	7.76
Mn (Cool)		0.0025	0.044	0.023±0.016	0.045			
Mn (Overall)		0.0025	0.044	0.021±0.003	0.042			
Fe (Warm)	mg/L	0.01	0.343	0.057±0.096	0.34	3 (Iran)	0.5	3.88
Fe (Cool)		0.0207	0.288	0.116±0.103	0.3			
Fe (Overall)		0.01	0.343	0.08±0.023	0.288			
Hg (Warm)	mg/L	0.0003	0.0035	0.00084±0.00091	0.003	0.01 (EPA)	1	7.76
Hg (Cool)		0.0003	0.001	0.00075±0.00022	0.00104			
Hg (Overall)		0.00026	0.0035	0.00081±0.00016	0.00098			
As (Warm)	mg/L	0.0006	0.0034	0.0021±0.0009	0.00342	0.1 (Iran)	1	7.76
As (Cool)		0.0007	0.0021	0.0013±0.00041	0.0022			
As (Overall)		0.0006	0.0034	0.0018±0.00020	0.00325			
Cd (Warm)	mg/L	0	0.003	0.00041±0.00091	0.00301	0.05 (Iran)	1	7.76
Cd (Cool)		0	0.0038	0.00076±0.00134	0.0046			
Cd (Overall)		0	0.0038	0.00055±0.00026	0.00381			
DO (Warm)	mg/L	2.87	7.4	5.766±1.318	7.3	2 (Iran)	0.5	3.88
DO (Cool)		5.098	6.21	5.541143±0.401	6.3			
DO (Overall)		2.87	7.4	5.678±0.246	6.81			
COD (Warm)	mg/L	14	203	103.82±63.653	200	200 (Iran)	0.1	0.78
COD (Cool)		32	200	104.71±58.131	210			
COD (Overall)		14	203	104.17±14.095	200			
BOD_5_ (Warm)	mg/L	8.1	107	52.518±32.198	104	100 (Iran)	0.1	0.78
BOD_5_ (Cool)		16.8	88	49.429±27.869	88.5			
BOD_5_ (Overall)		8.1	107	51.317±7.017	89.5			
NO_3_-N(Warm)	mg/L	3.79	46	24.067±13.444*	41*	5 (WHO)	0.1	0.78
NO_3_-N(Cool)		11.015	149.9	57.712±50.442*	165*			
NO_3_-N(Overall)		3.79		149.9	37.151±8.463*	91.58*			
TC (Warm)	N/100ml	20	2320	1014.82±1139.364*	2313*	1000 (Iran)	0.5	3.88	
TC (Cool)		24	2615	1725.28±1152.32*	2618*				
TC (Overall)		20	2615	1291.11±1165.88*	2437*				
FC (Warm)	N/100ml	15	1985	864.45±1071.53*	1980*	400 (Iran)	0.5	3.88	
FC (Cool)		6	1220	377.86±893.088*	1226*				
FC (Overall)		6	1985	675.22±1008.21*	2341*				
Helmith egg (Warm)	N/L	49	210	126.75±75.769*	208*	1 (Iran)	0.5	3.88	
Helmith egg (Cool)		20	164	66.35*±65	168*				
Helmith egg (Overall)		20	210	73.61*±96.125	215*				

* Values which did not meet standards.

Moreover, in order to get a closer CWQI to the actual quality of SWTP effluent, the authors decided to give weight to each parameter based on its importance in the agricultural irrigation. Thus, as Table 2 shows, each parameter has its specific multiplier and contribution value to calculation of CWQI.

## Results

**Figure 1:**
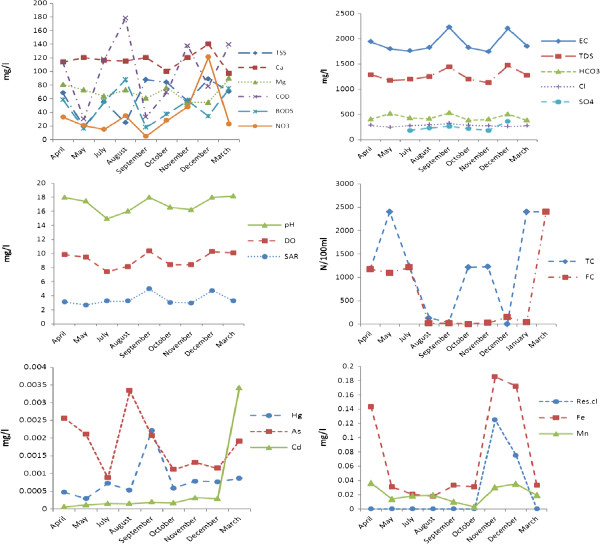
Mean variations of the measured parameters in SWWPTE.

After analyzing the samples collected from warm and cold seasons, the results shown in Table 2 were obtained. Besides, the meanvariations of the analyzed parameters are depicted in Figure 1.

**Figure 2:**
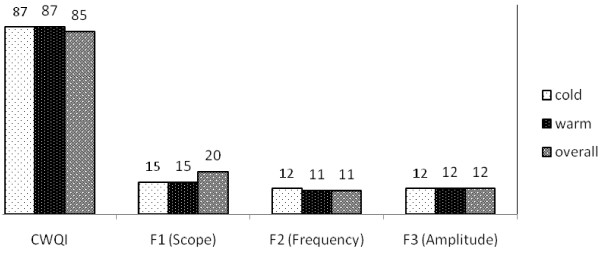
**The value of calculated CWQI, F**_**1**_**, F**_**2**_**, and F**_**3 **_**for physicochemical parameters.**

As noted above, to determine the quality of the SWTPE, the CWQI was used. Therefore, the CWQI was calculated in the warm, cold, and overall seasons for the physiochemical parameters. In addition, F_1_, F_2_, and F_3_ were separately calculated and the results are depicted in Figure 2.

Analysis of SWTPE shows that in the cold seasons, 4 physicochemical parameters (EC, Res. Cl, TSS, TDS, Mg, and NO_3_) failed from the defined objectives (Scope) and among these; two parameters (EC and NO_3_) had the highest failure to meet the objectives (Frequency). Also,NO_3_ had the most deviation from the desired objective (Amplitude). In the warm seasons, NO_3_, BOD_5_, COD,HCO_3_,TDS, TSS, and EC departed from their objectives and EC and TDS had the most frequency of failure. Besides, similar to the cold seasons, NO_3_ had the most deviation from its objective in warm seasons, as well.

**Figure 3:**
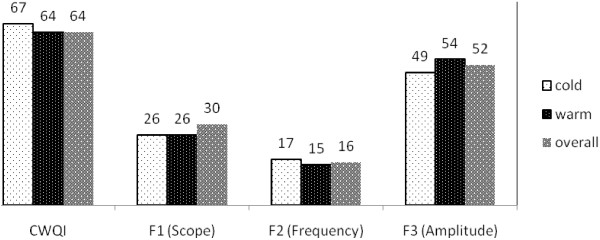
**The value of calculated CWQI, F**_**1**_**, F**_**2**_**, and F**_**3 **_**for physicochemical and microbial parameters.**

In all the cold and warm seasons, 9 parameters (EC, Res. Cl, TSS, TDS, Mg, NO_3_, BOD, COD, and HCO_3_) failed to meet their objectives over the sampling period. Among these parameters, similar to warm and cold seasons, the electrical conductivity had the most frequency of failure and NO_3_ had the most deviation from its objectives. Also, CWQI was calculated by applying the microbial parameters along with the physicochemical parameters (Figure 3). In this situation, the helminthes egg, instead of NO_3_, had the most deviation from its objective and fecal coliform as well as total coliform had failures to meet their objectives in warm, cold, and all the seasons together.

## Discussion

There are two components for evaluating the quality of water resources: 1) measurement of water quality variables and 2) comparison of values to benchmarks, such as guidelines or objectives. However, assessment of the quality variable by variable and objective by objective is quite a difficult task [23]. Therefore, a method which combines all the variables and represents a final value as the quality index could be used as a management tool for decision makers [27,28]. The CWQI is a science-based communication tool which tests multivariable water quality data versus water quality objectives specified by the users [23]. This tool also simplifies the reporting of water quality data to both technical and non-technical individuals [26]. Thus, due to the advantages of CWQI, in order to assess SWTP effluent quality for agricultural irrigation, this was used in the present study. According to Figure 2 and Table 1, physicochemical quality of SWTPE in warm and cold seasons is in the good range and, consequently, the physicochemical quality of the SWTPE rarely falls from the desired quality. According to the obtained results and Table 2, it can be concluded that TDS, EC, and NO_3_ have the largest contribution to the decline of CWQI in cold, warm, and all the seasons together. EC and TDS are the most important parameters related to the water resources salinity [29]. Some studies have shown that using wastewater for irrigation can increase soil salinity [6,8,11]. In the current study, mean and 90% cumulative probability of EC and TDS, which were the main factors of decrease in CWQI, were exceeded from the standards; therefore, this effluent could increase the irrigated soil salinity in future. In general, when the total soluble salt reaches an excessive concentration in the irrigated soil, water uptake by plant is reduced due to osmotic effect and this situation leads to a phenomenon called "osmotic desiccation" which can reduce the harvest [12,29,30]. On the other hand, increasing salinity reduces organic complex for most metals, which induces the displacement of metal in the solid phase with the soil solution and this can pollute the aquifers [31]. Generally, the salinity of WWTPE is high and the conventional treatments cannot reduce the salinity to the desired values; thus, just the advanced treatments which increase the cost of water reuse are necessary [12]. Overall, there are some options for controlling SWTPE salinity. For instance, in order to prevent soil salinization by SWTPE irrigation, enough drainage and leaching could be applied [30,32]. Also, if the salinity of the effluent is higher than the cultivated plant tolerance threshold, salinity could be reduced to the desired level by mixing the effluent with fresh water [13]. In the present study, the mean and 90% cumulative probability of nitrogen in warm, cold, and all the seasons together were far from the WHO standard (5mg/l). Some studies have shown that using untreated wastewater can increase soil nitrogen [10]. Although using wastewater treatment plants effluent for irrigation can be as significant source of valuable nutrients like nitrogen [14], it should be considered that large quantities of nitrogen in the effluent could be unfavorable for plant growth [11]. On the other hand, nitrate is highly soluble and by leaching phenomena, the nitrate concentration could increase in groundwater and consuming this water by the infants could lead to methemoglobinemia [33]. Hence, it seems that advanced treatments are necessary in order to reduce the SWTPE nitrate to the guidelines value. As can be seen in Figure 3, when microbial parameters were applied for calculating CWQI, the value of this index fell dramatically (from 85 to 64 in all the seasons together) and, thus, the quality of the effluent was located in marginal situation. Figure 3 also shows that the quality of SWTPE in the cold seasons was better than warm seasons, which could be due to the lower levels of microbial indicators in the cold seasons. In fact, the mean of fecal coliform and helminthes egg in cold seasons were respectively 486 and 61 units less than the warm seasons. Just the mean of total coliform in cold seasons was greater than the warm seasons, which might result from more precipitation in the cold period, washing the pathways, and progression of the washed coliforms in to the SWTP. Many studies have shown that the microbial pollution in the recycled effluent could contaminate the soil as well as the crops and develop the risk of disease in both consumers and the farm workers. AL-Laham et al. showed that irrigating tomato by an effluent with high microbial index can cause contamination on fruit scar [7]. In another study, Forslund et al. showed that using effluents for irrigation of potatoes could increase the risk of gastroenteritis diseases for farm workers [34]. Palese et al. also conducted a study and concluded that the reuse of wastewater for irrigation could increase the soil microbial load, although after a day, the contamination of the soil had greatly reduced [2]. In separate studies, Habbari et al. and Ensink et al. showed that the prevalence of parasitic infections was quite high among the populations exposed to the areas irrigated with recycled wastewater [19,20]. Therefore, considering the high levels of microbial indicators (Fecal coliform, Total coliform, and Helminthes egg) in SWTPE, it seems that using this water resource for irrigation could cause health problems for both the crops consumers and the farm workers and in order to reduce the microbial load in this wastewater treatment plant, some additional treatment, such as sand filtering followed by UV disinfection, is recommended. Bakopoulou et al. evaluated four wastewater treatment effluents for agricultural irrigation and showed that the wastewater treatment plant which used the advance treatment (sand filtering and UV disinfection) not only had a better microbial situation, but its physicochemical parameters were also in a better status compared to the other WWTPs [35]. Furthermore, applying management measures can control the health risk to some extent; for example, subsurface irrigation can be used in order to reduce the exposure of workers and crops to the recycled water. Stopping the irrigation few days before harvesting the crops [2,36], planting the crops in depths of the soil, putting nets under the trees in order to prevent the crops from falling on the ground and contamination of the product [2], and cooking the harvested crops before consumption [7], are other management practices which can bring down the risk of recycled wastewater for irrigation. As Table 2 depicts, mean and 90% cumulative probability of BOD_5_, TSS, HCO_3_, Cl, SO_4_, Ca, Mg, SAR, DO, Mn, Hg, Fe, As, and Cd completely fulfilled the standards, which shows the desirable efficiency of the treatment of the physicochemical parameters in SWTPE which is confirmed by the obtained CWQI values (Figure 2). Therefore, it seems that if the problems related to the microbial load in SWTPE be resolved, even with the current situation of the physicochemical parameters which could not meet the objectives (NO_3_, TDS, and EC), the final quality of SWTPE for agricultural irrigation will be favorable.

## Conclusion

The present study evaluated the SWTPE quality for agricultural irrigation by measuring the physicochemical and microbial parameters and then calculating the CWQI. The results showed that the effluent physicochemical quality was appropriate for irrigation; however, considering the microbial parameters, the quality of the effluent reduced dramatically which shows that the pathogens in this effluent can be a threat to the public health. Therefore, in order to protect the health of the consumers and the farm workers, advanced treatments, such as sand filtration and UV disinfection, are recommended.

## Competing interest

This work was financially supported by vice chancellor for research affairs of Shiraz University of Medical Sciences, Shiraz, Iran (contract no. 6029).

## Authors’ contributions

MAB participated in the data gathering, design of study, coordinated activities and revised manuscript. SN participated in the design of the study, final revised of manuscript and intellectual helping for analyzing of data. BD performed data collection, carried out statistical and technical analysis of data, participated in design of study and drafted manuscript. All authors read and approved the final manuscript.
